# Zeb1 and SK3 Channel Are Up-Regulated in Castration-Resistant Prostate Cancer and Promote Neuroendocrine Differentiation

**DOI:** 10.3390/cancers13122947

**Published:** 2021-06-12

**Authors:** Fanny Bery, Mathilde Cancel, Maxime Guéguinou, Marie Potier-Cartereau, Christophe Vandier, Aurélie Chantôme, Roseline Guibon, Franck Bruyère, Gaëlle Fromont, Karine Mahéo

**Affiliations:** 1 N2C UMR 1069, University of Tours, INSERM, F-37032 Tours, France; fanny.bery@etu.univ-tours.fr (F.B.); mathilde.cancel@univ-tours.fr (M.C.); maxime.gueguinou@univ-tours.fr (M.G.); marie.potier-cartereau@univ-tours.fr (M.P.-C.); christophe.vandier@univ-tours.fr (C.V.); aurelie.chantome@univ-tours.fr (A.C.); R.GUIBON@chu-tours.fr (R.G.); gaelle.fromont-hankard@univ-tours.fr (G.F.); 2 Department of Oncology, CHRU Bretonneau, CEDEX 9, F-37044 Tours, France; 3 CHRU of Tours, Department of Pathology, N2C UMR 1069, University of Tours, INSERM, CEDEX 9, F-37044 Tours, France; 4 CHRU of Tours, Department of Urology, CEDEX 9, F-37044 Tours, France; f.bruyere@chu-tours.fr

**Keywords:** prostate cancer, neuroendocrine differenciation, epithelial to mesenchymal transition, calcium signaling

## Abstract

**Simple Summary:**

Currently, neuroendocrine prostate cancers remain fatal, so it is crucial to better understand mechanisms of resistance to hormone therapy driving this phenotype. We have shown that Enza, a new generation hormone therapy, promotes prostate cancer cells neurodifferentiation by activating a positive feedback loop between the key transcription factor of epithelial to mesenchymal transition Zeb1 and the calcium-sensitive potassium channel SK3. These two actors are overexpressed in patients with neuroendocrine castration-resistant prostate cancer. Targeting SK3 channel by Ohmline, a synthetic ether lipid, inhibits neuroendocrine differentiation of prostate cancer cells, which opens new therapeutic prospects for neuroendocrine prostate cancers.

**Abstract:**

Therapeutic strategies for metastatic castration-resistant prostate cancer aim to target androgen receptor signaling. Despite initial survival benefits, treatment resistance invariably occurs, leading to lethal disease. Therapies targeting the androgen receptor can induce the emergence of a neuroendocrine phenotype and reactivate embryonic programs associated with epithelial to mesenchymal transition. We recently reported that dysregulation of the calcium signal can induce the transcription factor Zeb1, a key determinant of cell plasticity during tumor progression. The aim of this study was to determine whether the androgen receptor-targeted treatment Enzalutamide could induce dysregulation of the calcium signal involved in the progression toward epithelial to mesenchymal transition and neuroendocrine differentiation, contributing to therapeutic escape. Our results show that Zeb1 and the SK3 potassium channel are overexpressed in vivo in neuroendocrine castration-resistant prostate cancer and in vitro in LNCaP cells neurodifferentiated after Enzalutamide treatment. Moreover, the neuroendocrine phenotype is associated with a deregulation of the expression of Orai calcium channels. We showed that Zeb1 and SK3 are critical drivers of neuroendocrine differentiation. Interestingly, Ohmline, an SK3 inhibitor, can prevent the expression of Zeb1 and neuroendocrine markers induced by Enzalutamide. This study offers new perspectives to increase hormone therapy efficacy and improve clinical outcomes.

## 1. Introduction

The initial treatment for metastatic prostate cancer (PCa) is androgen deprivation therapy (ADT) since the androgen receptor (AR) signaling axis is the major driver of PCa cells. ADT is initially effective in controlling the disease, which unfortunately progresses over a few months or years to castration-resistant prostate cancer (CRPC). The introduction of a new generation of anti-androgens, including the AR targeted therapy Enzalutamide (Enza), has improved patient survival, but the disease inevitably relapses and remains fatal [1,2].

The emergence of a neuroendocrine (NE) phenotype is observed in between 10 and 20% of CRPC (CRPC-NE) and is associated with a poor prognosis when compared to CRPC that retains an adenocarcinoma phenotype (CRPC-adeno). NE prostate cancer (NEPC) is an aggressive disease that rarely arises de novo, but often emerges after hormonal manipulation by ADT or AR targeted therapy [3]. Post-treatment NE differentiation (NED) reflects tumor adaptation by epithelial plasticity. NE cells exhibit the morphological and physiological characteristics of both neural and endocrine differentiation. NE cells usually lack AR and are likely to be androgen-independent. The cytoplasm of NE cells is enriched with secretory granules containing neuropeptides and hormones, such as chromogranin A, synaptophysin and neuron-specific enolase (NSE) which can exercise a paracrine effect on neighboring non-NE cancer cells to regulate their proliferation [4].

Hormone therapy can also reactivate in PCa cells embryonic programs associated with epithelial-to-mesenchymal transition (EMT). The EMT process is correlated with aggressive behavior and resistance to treatment observed in advanced PCa [5,6]. EMT increases the migratory and invasive capacities of cancer cells through the acquisition of a mesenchymal-like phenotype [7]. Among EMT markers, we previously demonstrated the clinical significance of the transcription factor Zeb1 (zinc finger enhancer-binding protein 1). Indeed, in human PCa, Zeb1 expression increases according to the disease progression and is associated with decreased survival [8]. Zeb1 is a key EMT transcription factor that promotes multidrug resistance, proliferation, and metastasis formation [7,9]. Previous studies have reported a negative feedback loop between AR and Zeb1. Indeed, AR inhibition by Enza enhances the expression of Zeb1, which on the other hand is reduced when AR is activated by dihydrotestosterone (DHT) [10,11]. Other studies reported that EMT markers, including Zeb1, are induced in LNCaP (Lymph Node Carcinoma of the Prostate) cells cultured with charcoal-stripped serum, and a xenograft model of PCa cells after castration [11].

We previously reported deregulation of calcium homeostasis, inducing in PCa cells in both the EMT process and the NED. Indeed, we demonstrated that calcium signaling plays a major role in early EMT events. We identified a new positive feedback loop between Zeb1 and the calcium-activated potassium channel SK3, that promotes calcium entries and cellular migration [12,13]. This signaling pathway was highlighted in PCa cells and human PCa explants treated by EMT inducers (TGFβ and hypoxia). We also reported that the Calcium-Sensing Receptor is a marker and a driver of NED in PCa [14]. Others have shown that the voltage-gated Ca^2+^ channel CaV3.2 is overexpressed in NEPC, together with an increase in calcium current density [15].

In the present study, we aimed to determine whether Enzalutamide could induce dysregulation of calcium homeostasis involved in the progression toward NED and EMT and whether targeting the SK3 channel could prevent this progression and therefore result in therapeutic escape. To this purpose, we used tissue microarrays containing human PCa tissue samples, PCa cell lines, and human PCa tissue explants, a model that preserves both cancer cell heterogeneity and microenvironment.

## 2. Materials and Methods

### 2.1. Patients and Tissues

#### 2.1.1. For Tissue Micro-Array Construction

Clinically localized cancer samples (CLC) (*n* = 220) were obtained from patients treated by radical prostatectomy for localized PCa at Tours University Hospital. Fifty cases of metastatic castration-resistant prostate cancers (CRPC) were selected from patients treated with androgen deprivation therapy (ADT). Tissues were collected either by transurethral resection, performed because of lower urinary tract symptoms associated with local tumor progression, or by biopsy from a metastatic site. After 2 consecutive rises in PSA, with serum testosterone under castration level (50 ng/dL), hormonal relapse was defined. Cases with prominent NE cell differentiation were excluded from this group and classified as treatment induced NEPC. Sixteen cases of treatment induced NEPC samples were obtained by transuretral resection or by biopsy from a metastatic site. The diagnosis of NEPC has been established in the case of a prominent NE cell population, identified on morphological criteria (small cells), and with positive immunostaining for either chromogranin or synaptophysin. Patients and tissue characteristics are specified in Table S1 (Supplementary Materials).

#### 2.1.2. For Culture of Human PCa Tissue Slices

PCa tissue samples were obtained from 4 patients undergoing radical prostatectomy for PCa. Patients were 58 to 71 years old, with ISUP score 2 (*n* = 2), 4 (*n* = 1) or 5 (*n* = 1). tumors were staged pT2 (*n* = 1) or pT3 (*n* = 3).

Written informed consents were obtained from patients following the requirements of the medical ethic committee of our institution (Comité de Protection des Personnes (CPP) de Tours—Région Centre Ouest I) (ethic code DC-2014-2045) (years between 2003–2018).

### 2.2. TMA Construction

TMAs (tissue micro-arrays) were constructed using formalin-fixed paraffin-embedded tissue samples. Original slides stained with hematoxylin-eosin were reviewed using the 2017 pTNM classification and the 2014 modified “Gleason” system, i.e., ISUP score. For each case, 3 cores (0.6 diameters) were transferred from the selected areas to the recipient block, using a TMA workstation (Manual Tissue Arrayer MTA Booster, Alphelys, France). Serial 3 µm sections of the TMA blocks were used for immunohistochemistry. One section on ten was stained with hematoxylin-eosin to check that the cores adequately represented diagnostic areas.

### 2.3. Immunohistochemistry on TMA, Cultured PCa Tissue Slices and Cell Pellets

Slides were deparaffinized, rehydrated, and heated in citrate buffer pH 6 for antigenic retrieval. After blocking for endogenous peroxidase with 3% hydrogen peroxide, the primary antibodies were incubated. The panel of primary antibodies included Zeb1 (Abnova, Taipei, Taiwan, Clone 4C4, dilution 1/500, 1 h), SK3 (LSBio, Seattle, WA, USA, dilution 1/200, 1 h), Orai1 (Lifespan, Providence, RI, USA, LS-C94375, dilution 1/4000, 1 h), Orai2 (Sigma prestige, St. Louis, MO, USA, HPA065937ph6, 1/100, 1 h), NSE (Cellmark, Gothenburg, Sweden, 760-47-85, 16 min), AR (Roche, Basle, Switzerland, clone SP107, 1/1, 30 min), and the proliferation marker Ki67 (DakoCytomation, clone 39-9, 1/50, 30 min). Immunohistochemistry was performed with either the automated BenchMark XT slide stainer (Ventana Medical Systems Inc., Oro Valley, AZ, USA) using OptiView Detection Kit (Ventana Medical Systems Inc.) or manually (for Zeb1, SK3, Orai1, Orai2) using the streptavidin-biotin-peroxidase method with diaminobenzidine as the chromogen (Kit LSAB, Dakocytomation, Glostrup, Denmark). Slides were finally counterstained with haematoxylin. Negative controls were obtained after omission of the primary antibody or incubation with an irrelevant antibody.

Scoring of antibody staining in TMA: The slides were analysed by 2 observers in a blinded fashion. Zeb1 was expressed as a percentage of total cancer cells. For SK3, Orai1 and Orai2, a semi-quantitative score was attributed, based on the intensity of staining: 0 (no staining), + (moderate staining), ++ (strong staining).

In case of inter-observer variability (different categories in the case of categorical data or variability more than 10% in the case of continuous data), which occurred in <5% of cases, slides were rescored to reach consensus.

Immunohistochemistry on cell pellet sections from LNCaP cells was performed using the same antibodies and technics as for the TMA sections.

### 2.4. Cell Lines and Products

PCa cells lines LNCaP (CRL-1740), and second-generation LNCaP subline: C4-2 (CRL-3314) and C4-2B (CRL3315) (B for Bone Metastatic) [16] were purchased from American Type Culture Collection (ATCC). LNCaP cells were cultured in Roswell Park Memorial Institute medium containing D-Glucose (4.5 g/L), HEPES Buffer (2383 g/L), L-Glutamine, Sodium Bicarbonate (1.5 g/L), sodium pyruvate (110 mg/L) (RPMI, A1049101, Lonza, Levallois-Perret, France) and supplemented with 10% fetal bovine serum (FBS) (CVF5VF00-01, Eurobio, Les Ulis, France) and 1% penicillin-streptomycin (PS). C4-2 and C4-2B cells are cultured in RPMI medium (BE12-702F, Lonza, Levallois-Perret, France) supplemented with 5% FBS and 1% PS. Cells were maintained in a 37 °C humidified incubator with 5% CO^2^.

The non-steroidal AR inhibitor, Enza (PHB00235) and the SK3 positive modulator CyPPA (Cyclohexyl-[2-(3,5-dimethyl-pyrazol-1-yl)-6-methyl-pyrimidin-4-yl]-amine) were purchased from Sigma-Aldrich (St-Quentin Fallavier, France). 1-Ohexadecyl-2-O-methyl-sn-glycero-3-lactose (Ohmline) was synthetised as previously described [17]. For one-week treatments, cells were treated 3 times per week with Enza, Enza+Ohmline or CyPPA.

### 2.5. Cell Line Pellets for Immunohistochemistry

Cells were washed with a Phosphate Buffered Saline solution (PBS) (CS1PBS00-01, Eurobio, Les Ulis, France), and were harvested by using TrypLE Express (12605-028, Gibco, MA, USA). Centrifugation at 1500 rpm for 5 min was performed to get a cell pellet, which was subsequently fixed in formalin, embedded in paraffin, and cut in 3 µm sections.

### 2.6. RNA Extraction and Quantitative Real-Time PCR

The Nucleopsin RNA kit (Macherey–Nagel, Hoerdt, France) was used according to the manufacturer’s protocol to extract total RNA. To determine RNA concentration and purity, absorbance at 230, 260, and 280 nm were measured with spectrophotometer EpochTM (Biotek Instruments, Inc., Colmar, France). Reverse transcription was carried out to obtain cDNA at 50 ng/µL from RNAs with RT kit (PrimeScriptTM RT Reagent, Perfect Real Time, Takara, Saint Quentin Yvelines, France) in a LabCycler (Sensoquest, Germany) at 37 °C for 17 min followed by 85 °C for 5 min. For each reaction, SYBR Green mix (RR420L, Takara) containing a double-strand DNA-binding dye was mixed with specific primers (0.5 µM) and cDNA (50 ng/µL). Each condition was performed in triplicate. The quantitative PCR was performed using CFX ConnectTM BIORAD and analyzed with Biorad CFX Maestro software. The ΔΔCT method was used to calculated relative levels of mRNA which were normalized to the housekeeping gene hypoxanthine phosphoribosyltransferase (HPRT) and TATA-binding protein (TBP). Primers used for quantitative real-time PCR are available in Table S2 (Supplementary Materials).

### 2.7. Transfection Assays

LNCaP cells used for transfection were previously treated (or not) for 1 week with Enza (10 µM). Next, LNCaP cells were cultured in a 6-well plate in a medium culture at a density of 250,000 cells per well. The next day, the transfection was performed in the medium for 96 h: Cells were incubated with the siRNA directed against Zeb1 and lipofectamineTM RNAiMAX (10514953, Fisher Scientific TM, Illkirch, France). On the next day, the medium was refreshed. siRNA was used at the final concentration of 10 nM. siRNA sequences are available in Table S3 (Supplementary Materials).

### 2.8. Cell Viability MTT Assay

MTT (3-(4,5-dimethylthiazol-2-yl)-2,5-diphenyl tetrazolium bromide) assay allows live-cell numeration by quantifying their mitochondrial activity. The tetrazolium salt MTT (3-(4,5-dimethylthiazol-2-yl)-2,5-diphenyl tetrazolium bromide) is reduced to formazan by the mitochondrial succinate dehydrogenase of living cells, generating a purple coloration. A total of 50,000 LNCaP were seeded in a 24-well plate and treated (or not) with Enza (10 µM) for 24 h, 72 h, or 1 week. Then, after cells incubation with MTT for 1 h at 37 °C, DMSO (dimethylsulfoxide) was added to dissolve formazan crystals. Each condition was performed in triplicate and absorbance was quantified with a spectrophotometer at 570 nM.

### 2.9. Culture of Human PCa Tissue Slices (Organotypic Culture)

Immediately after surgery, samples of 4 to 5 mm were dissected aseptically and slices were cut with a vibratome into 6–8 slices per sample, as previously described [18]. Slices were incubated in 6-well plates containing 2 ml of Dulbecco Modified Eagle Medium supplemented with 10% FBS and DHT (1 nM) (Sigma, D073) in an incubator at 37 °C and 5% CO^2^. Explants were treated for 5 days with Enza (10 µM) in the presence (or not) of Ohmline (1 µM) and then fixed with 4% buffered formalin and used for immunohistochemical staining.

### 2.10. Cytosolic Calcium Measurements

Cytosolic calcium concentration was measured by using the ratiometric fluorescent dye Fura-2-AM purchased from Molecular Probes (1 h incubation, 1 µg/mL). Fluorescence emission was measured at 510 nm with an excitation light at 340 and 380 nm.

For SOCE (store-operated calcium entry) experiments, we used the FlexStation 3 multi-mode microplate reader with automated pipetting. LNCaP cells were seeded in a 96-well plate previously coated with fibronectin (1/200 dilution). The next day, after 1 h incubation with the Fura-2-AM (acetoxymethyl ester) probe, cells were placed in free calcium PSS (physiological saline solution) (composition in Table S4 of supplementary materials) and after a stabilizing time (100 s), an injection of thapsigargin (Tg 5 µM) was realized leading to endoplasmic reticulum calcium depletion. When cytosolic calcium concentration returns to baseline (500 s), PSS with CaCl2 (2 mM) was added. Each condition was performed in quadruplicate. Analyses were performed using the SoftMax Pro software (5.4.6 version, Molecular Devices, San Jose, CA, USA).

For constitutive calcium entry (CCE), we used a fluorescence spectrophotometer (F-2710, Hitachi High Technologies America, Inc., Pleasanton, CA, USA). After 1 h incubation with the Fura-2-AM probe, cells were harvested and cells pellets were obtained. Then, cells were placed in free calcium PSS and after a stabilizing time (30 s), PSS with CaCl2 (2 mM) was added. Each condition was performed in triplicate.

### 2.11. Luciferase Assay

For the luciferase assay, 55,000 cells were seeded in 24-well plates in triplicate for each condition. The transfection was performed 48 h later with 0.48 µL of TransIT-2020 reagent (Mirus, Madison, United States), 5 ng of pRL-TK (Promega, Madison, WI, USA), 50 ng of pGL4.17-KCNN3, and 100 ng of pCIneo-ZEB1 or pCIneo vector control. Insertion of KCNN3 (Potassium Calcium-Activated Channel Subfamily N Member 3, SK3 channel encoding gene) promoter in pGL4.17 vector has been described previously [12]. pCIneo-ZEB1 was kindly provided by Marc Stemmler (Alexander University of Erlangen-Nürnberg, Erlangen, Germany). Cells were lysed 48 h after transfection with passive lysis buffer (Promega) and luciferase assays were measured with Dual-Luciferase^®^ Reporter Assay System (Promega) according to the manufacturer’s instructions.

### 2.12. Statistical Analysis

Statistical analysis was carried out using Prism 6 software (GraphPad Software, San Diego, CA, USA). For comparison of two groups with continuous variables, the Mann–Whitney test was used. When there were more than two groups, a Kruskal–Wallis test was performed, followed by a Dunn post-test allowing the means to be compared in pairs. Results were considered as significant when the probability was less than or equal to 0.05 and was expressed as mean ± SEM (as indicated in figure legends).

## 3. Results

### 3.1. The Expression of Zeb1, SK3 and Orai2 Is Increased in PCa Samples from Patients with CRPC (Adeno and NE)

**Figure 1:**
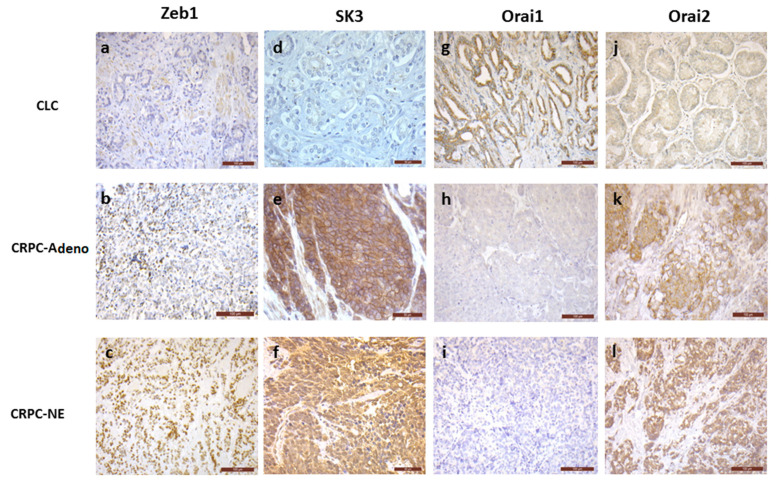
Representative images of immunohistochemical staining of Zeb1 (**a**–**c**), SK3 (**d**–**f**), Orai1 (**g**–**i**) and Orai2 (**j**–**l**), in human PCa samples. In CLC, most of the cases showed no Zeb1 (1a, bar 100 µm) and SK3 (1d, bar 50 µm) expression, while Zeb1 and SK3 positive staining was found in the majority of CRPC-adeno and CRPC-NE samples (*p* = 0.01 to < 0.0001) (1bc, bar 100 µm, 1ef, bar 50 µm). Orai1 was expressed in most of CLC cases (1g, bar 100 µm), without any expression in the majority of CRPC–adeno and CRPC-NE samples (1hi, bar 100 µm) (*p* < 0.0001 and 0.004). No Orai2 staining was found in more than 40% of CLC samples (1j, bar 100 µm), while Orai2 was expressed in more than 80% of CRPC-adeno and CRPC-NE cases (1kl, bar 100 µm) (*p* = 0.02 and 0.0004).

We investigated, in vivo, the expression of Zeb1, SK3 (a potassium channel), and Orai1/2 (two calcium channels) in tumor samples obtained from 220 patients with hormone naïve clinically-localized cancer (CLC), 50 patients with adenocarcinoma CRPC (CRPC-adeno), and 16 patients with NE CRPC (CRPC-NE). Results are summarized in Table 1 and Figure 1.

Briefly, Zeb1 expression is increased in CRPC-adeno (66% of positive cases) and in CRPC-NE (93% of positive cases), when compared to CLC (34% of positive cases) (*p* = 0.01 and <0.0001, respectively). The percentage of cases expressing SK3 is higher in CRPC-adeno (73% of positive cases) and CRPC-NE (62% of positive cases) than in CLC (27% of positive cases) (*p* < 0.0001). Similar results were observed for Orai2, with increased expression in CRPC-adeno (80% of positive cases) and CRPC-NE (86% of positive cases), compared to CLC (60% of positive cases) (*p* = 0.02 and 0.0004, respectively). In contrast, cancer samples expressing Orai1 dramatically decreased in CRPC-adeno (4% of positive cases) and in CRPC-NE (21% of positive cases) when compared to CLC (64% of positive cases) (*p* < 0.0001 and 0.004, respectively).

### 3.2. AR Inhibition by Enza Induces LNCaP Neurodifferentiation

**Figure 2:**
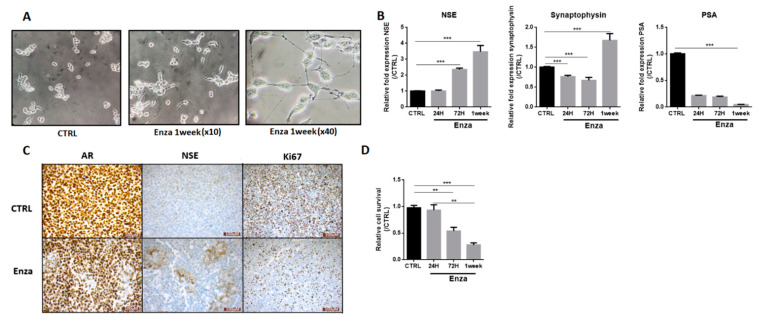
AR inhibition induces LNCaP neurodifferentiation. (**A**): Enza induces cytoplasmic extension in LNCaP cells. Pictures were obtained with an optical microscope (magnification ×10 or ×40); (**B**): Enza first increases neuronal marker expression, later NE markers, and inhibits PSA expression. LNCaP were treated with Enza (10 µM) for 24 h, 72 h, and 1 week. qPCR results are normalized to the control condition and are expressed as mean ± S.E.M. The statistical differences are indicated: *** *p* < 0.001 (Kruskal–Wallis; post-test: Dunn) *n* = 3; (**C**): Enza treatment for 7 days decreases nuclear AR expression in cell foci expressing NSE and drastically decreases LNCaP proliferation. AR, NSE and Ki67 protein staining were obtained by immunohistochemistry; (**D**): Enza decreases cell proliferation. MTT assay was performed in LNCaP cells treated (or not) with Enza (10 µM) for 24 h, 72 h, and 1 week. Results are normalized to the control condition and are expressed as mean ± S.E.M. The statistical differences are indicated: ** *p* < 0.01; *** *p*< 0.001 (Kruskal–Wallis; post-test: Dunn) *n* = 3.

Androgeno-dependent LNCaP cells were treated with Enza, an AR inhibitor. As observed in Figure 2A, Enza treatment (1 week) induced changes in cell morphology with cytoplasmic extensions similar to neurite outgrowths. We evaluated at 24 h, 72 h, and 1 week the expressions of NE markers such as neuron-specific enolase (NSE), synaptophysin (Figure 2B) and chromogranin A. While chromogranin remained undetectable (data not shown), NSE mRNA levels increased from 2.4-fold and 3.5-fold at 72 h and 1 week following Enza treatment, respectively. Synaptophysin expression was little affected at 24 and 72 h exposure but was clearly increased from 1.7-fold after a 1-week treatment. Figure 2C shows that the Enza treatment (1 week) induced an increase in NSE protein expression together with a decreased nuclear expression of AR protein in the same foci. Note that the down-regulation of AR is a characteristic of NEPC [19]. Altogether these results showed that Enza promotes NE differentiation (NED).

As expected, Enza for 1 week induced a drastic decrease in the proliferation of LNCaP cells as observed by the Ki67 protein staining and MTT assay (−46% at 72 h and −73% at 1 week) (Figure 2C,D). The efficiency of Enza on the AR-signaling pathway is attested by a strong reduction of Prostate-Specific Antigen (PSA) mRNA level (which is a pivotal downstream target gene of the AR) in the first 24 h after treatment (Figure 2B).

We also tested the effect of Enza on C4-2 and C4-2B CRPC cell lines, which are hormone-independent sublines of LNCaP cells, and represent in vitro cell models of CRPC. NSE expression was induced, with a two-fold increase in mRNA levels in both cell lines (Supplementary Figures). These results suggest that Enza promotes the NED process at a more advanced stage of NED in LNCaP compared to LNCaP-derived CRPC cell lines. Indeed, unlike NSE, synaptophysin is a marker for well-differentiated NE tumors [20].

### 3.3. Zeb1 Is Required for Enza-Induced NED

**Figure 3:**
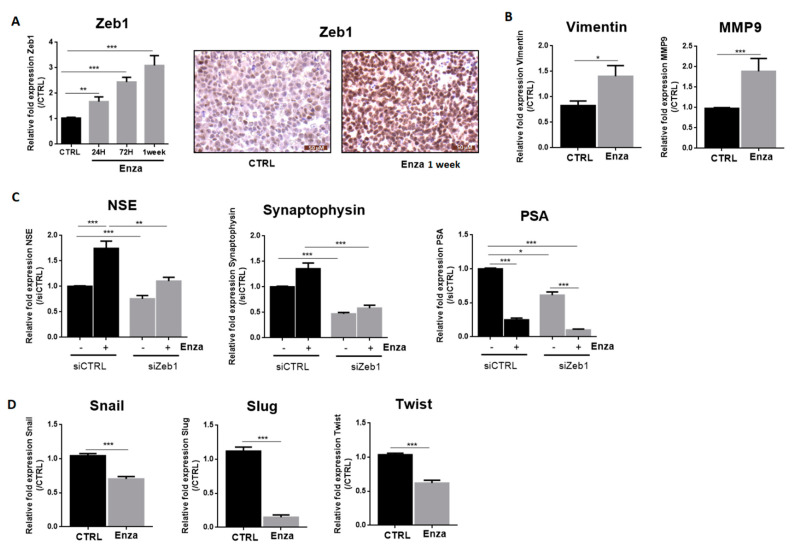
NED induced by AR inhibition depends on the EMT transcription factor, Zeb1. Graphs represent qPCR results and pictures represent immunohistochemistry results. All Enza treatments (10 µM) were performed for one week except in the cases indicated. (**A**,**B)**: Enza increases Zeb1 and its target genes expression; (**C**): Effects of Zeb1 inhibition on neuroendocrine markers and PSA expression. LNCaP cells were treated with Enza (10 µM) for 1 week and then used for transfection assay performed for 96 h with a siRNA directed against Zeb1; (**D**): Enza decreases the EMT transcription factors Snail, Slug and Twist. qPCR results are normalised to the control condition and are expressed as mean ± S.E.M. The statistical differences are indicated: * *p* < 0.05; ** *p* < 0.01; *** *p* < 0.001 (Mann–Whitney (**B**,**D**); Kruskal–Wallis; post-test: Dunn (**A**,**C**) *n* = 3 for all experiments except for transfection assays, *n* = 6.

Zeb1 mRNA expression is time-dependent and was markedly increased as it showed 1.7-fold, 2.4-fold and 3-fold increase at 24 h, 72 h, and 1-week treatment times, respectively, (Figure 3A). Immunohistochemical analysis of Zeb1 expression revealed strong nuclear staining in most treated cells (Figure 3A). Among the target genes of this transcriptional factor, we examined vimentin and MMP9 gene expression. As expected, Enza treatment increased mRNA MMP9 expression by about 1.8 and vimentin by 1.4 times (Figure 3B).

We considered whether Zeb1 was involved in the maintenance of NE features induced by Enza treatment. After inducing NED with 1 week of Enza treatment, cells were transfected with a siRNA against Zeb1. Interestingly, the reduction of Zeb1 expression completely reverses the Enza-driven NE phenotype, with NSE and synaptophysin expression returning to basal levels. Note that PSA expression remained repressed by Enza in Zeb1-transfected cells, suggesting that Zeb1 may be an interesting target to reverse NE differentiation without affecting the ability of Enza to inhibit the AR signaling pathway (Figure 3C). Concerning the other transcription factors involved in EMT, Enza treatment (1 week) led to a decreased mRNA level of Snail (−30%), Slug (−85%) and Twist (−40%) (Figure 3D).

### 3.4. Enza Treatment Affect SK3 and Orai Expression: Impact on Calcium Entry Pathways

We previously identified a positive feedback loop between Zeb1 and the SK3 potassium channel that contributes to prostate cancer cell migration [12,13]. The calcium-activated potassium channel SK3 is known to form complexes with Orai1 calcium channel to induce calcium entry that mediates an aggressive phenotype in human cancer cells [21]. Thus, we examined the effect of Enza on SK3, Orai1 and two other Orai family members, Orai2 and Orai3.

**Figure 4:**
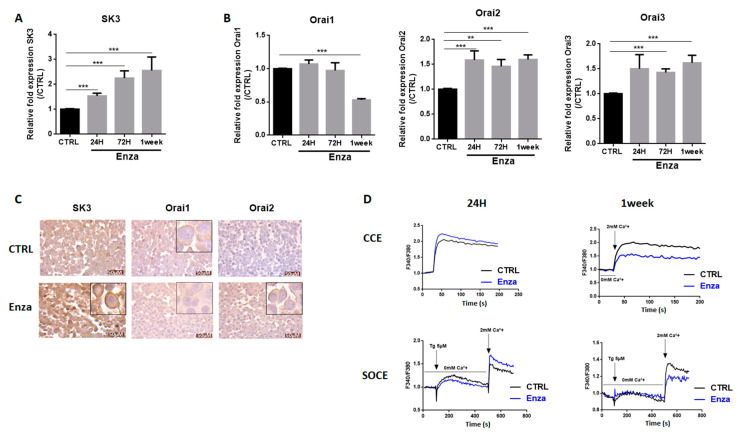
AR inhibition deregulates calcium signaling. (**A**,**B**): Enza first increases Orai2, Orai3, and SK3 expression and later, after a 1-week treatment decreases Orai1 expression. LNCaP cells were treated for 24 h, 72 h, and 1 week with the Enza (10 µM). qPCR results are normalized to the control condition and are expressed as mean ± S.E.M. The statistical differences are indicated: ** *p* < 0.01; *** *p* < 0.001 (Kruskal–Wallis; post-test: Dunn) *n* = 3; (**C**): Expression of SK3, Orai1 and Orai2 proteins in LNCaP cells treated with Enza for 1 week. Immunohistochemical staining was performed on cell pellets; (**D**): Two main calcium entry pathways (CCE and SOCE) were measured by fluorimetry with the Fura2-AM probe. LNCaP cells were treated with Enza (10 μM) for 24 h and 1 week. Results are represented by calcium curves illustration.

The expression profile of SK3 in Enza-treated cells is similar to that observed for Zeb1, i.e., a progressive increase in mRNA levels with treatment time (1.5-fold at 24 h; 2.2-fold at 72 h and 2.5-fold at 1 week) (Figure 4A). A strong expression of SK3 localized on the plasma membrane after 1 week of treatment was observed by immunohistochemical analyses (Figure 4C). Interestingly, the induction of Zeb1 and SK3 by Enza was also found in C4-2 and C4-2B cell lines (Supplementary Figures).

Concerning Orai channels, Enza treatment affected the expression of Orai1 but only after 1 week of treatment with a halving of mRNA levels and a clear decrease in membrane expression (Figure 4). In contrast, the expression of the Orai2 and Orai3 channels is increased after 24 h of treatment 1.5-fold. Interestingly, most of the Orai2 were found to be localized on the plasma while the Orai2 plasma membrane staining in untreated cells is very weak.

Next, we investigated the consequences of Enza treatment on the two main calcium entry pathways, the store-operated calcium entry (SOCE) and the constitutive calcium entry (CCE), which we found to be regulated by SK3 and Orai1 channels [21,22]. After 24 h of treatment, SOCE is increased by 20% (*p* < 0.01) and the CCE remained unchanged. However, after 1-week of treatment, SOCE and CCE are both inhibited by 30% (*p* < 0.001) and 32% (*p* < 0.001), respectively. Altogether, these results suggest that (i) SOCE amplification may be an initial event in the signaling pathway of the Enza-promoted NE process and (ii) the decrease in calcium entries explained by the loss of Orai1 expression, could occur in cells in advanced NE phenotype.

### 3.5. Neuroendocrine Effects of Enza Is Driven by SK3/Zeb1 Pathway

**Figure 5:**
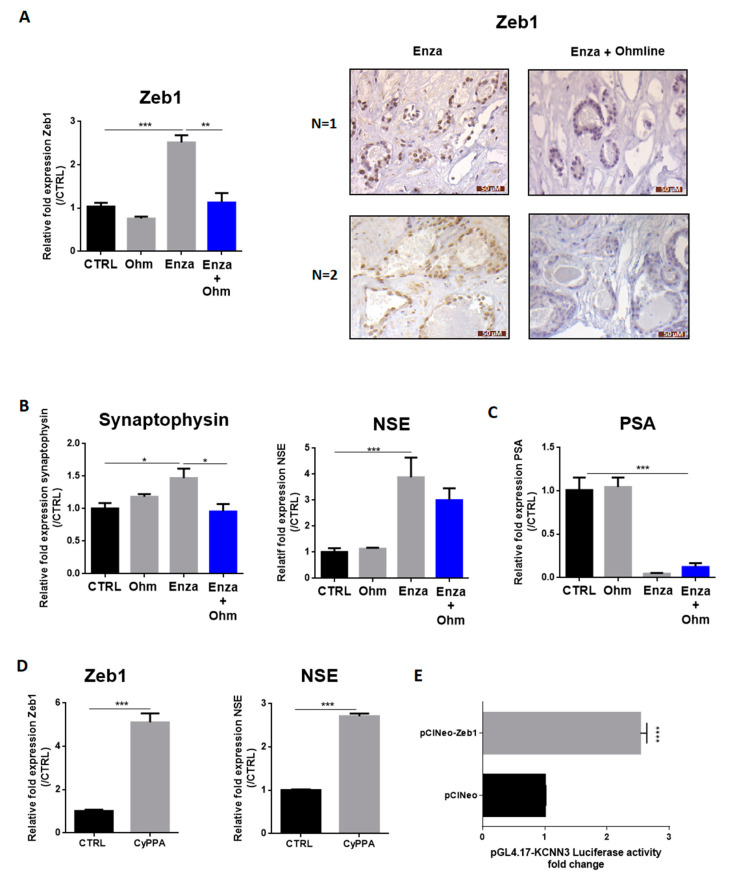
AR inhibition-induced NED depends on the SK3 potassium channel. (**A**–**C**): LNCaP cells were treated by Enza (10 µM) alone, with Ohmline (Ohm) alone or co-treated with Enza (10 µM) + Ohmline (1 µM) for 1 week. Immunohistochemical staining of Zeb1 (A) was performed in human PCa samples treated with Enza (10 µM) alone for 5 days or co-treated with Enza (10 µM) + Ohmline (1 µM) for 5 days; (**D**): LNCaP cells were treated (or not) with CyPPA (10 µM) for 1 week. Graphs represent qPCR results and pictures represent immunohistochemistry results. qPCR results are normalized to the control condition and are expressed as mean ± S.E.M; (**E**): Overexpression of Zeb1 enhanced KCNN3 gene transcription in LNCap cells. LNCaP cells were co-transfected with pGL4.17-KCNN3, pRL-TK and pCIneo (control condition) or pCIneoZeb1. Dual-luciferase reporter assays were performed 48 h after transfection. Results are normalized to the control condition and are expressed as mean ± S.E.M. The statistical differences are indicated: * *p* < 0.05; ** *p* < 0.01; *** *p* < 0.001 (**A**–**C**: Kruskal–Wallis; post-test: Dunn) (**D**,**E**: Mann–Whitney), *n* = 3 for all experiments except for KCNN3 promotor activity *n* = 4.

In our previous studies, we highlighted a positive feedback loop between Zeb1 and the SK3 channel [12]. To determine the potential involvement of this pathway in Enza-induced NED, LNCaP cells were treated by Enza in presence of Ohmline, a specific inhibitor of the SK3 channel [17]. As observed in Figure 5, Ohmline prevented the induction of Zeb1 expression by Enza (Figure 5A) and the induction of synaptophysin and NSE by Enza (Figure 5B). However, Ohmline was unable to reverse the process when the cells are well neurodifferentiated (with neurites outgrowth and NE markers expression), after 1 week of the Enza treatment (Supplementary Figures). Enza in combination with Ohmline remained efficient as an AR inhibitor since a reduced PSA mRNA level was maintained. In addition, the activation of SKCa channels by CyPPA, induced a strong induction of Zeb1 (five-fold) and NSE marker (2.7-fold). To determine whether *KCNN3* (SK3 channel encoding gene) is a target gene of Zeb1, we monitored gene transcription from a promoter-luciferase reporter constructs in LNCaP-transfected with the pCIneo-Zeb1 plasmid, which allows constitutive transcription of Zeb1. Overexpression of Zeb1 increased *KCNN3* promoter activity (2.5-fold) (Figure 5E). Altogether, these results suggest the involvement of an SK3/Zeb1 pathway in the initial steps of the NED process.

We next investigated whether these findings could also be observed in clinical samples. Human PCa slices were treated with Enza in combination (or not) with Ohmline for 5 days (*n* = 4 patients). Indeed, we previously demonstrated that prostate tissue slices can be cultured for up to 5 days without necrosis and preserving physiological properties [18]. Zeb1 expression was analysed by immunohistochemistry. All tumors (*n* = 4) showed no Zeb1 staining at baseline, as the majority of clinically localized PCa. The Enza treatment led to induce Zeb1 expression in two of them, and the two others remained negative. The Ohmline treatment combined with Enza was able to prevent Zeb1 induction in both cases (Figure 5A). 

## 4. Discussion

AR targeted therapy used in metastatic PCa can lead to epithelial plasticity that enables adaptation of cancer cells, with the emergence of NE and EMT markers that contribute to treatment failure. We showed for the first time that the key EMT driver Zeb1 and the SK3 channel are highly expressed in most of the patients with CRPC, including CRPC-NE. Moreover, we observed a reshaping of the Orai in CRPC compared to CLC, with decreased Orai1 together with increased Orai2 expressions. Similar modulations were observed in LNCaP cells treated by Enza, an AR-targeted therapy widely used in CRPC patients. We demonstrated that the transcriptional factor Zeb1 is required in the NED process. Since Zeb1 regulates *KCNN3* (encoding SK3), these results strongly support the existence of a positive feedback loop between Zeb1 and the SK3 channel, involved in NED and induced by Enza. The lack of Zeb1 induction during Enza/Ohmline co-treatment in human PCa slices is an encouraging finding, opening new therapeutic perspectives with Ohmline being a promising compound to be developed as the first class of lipid-anticancer agents.

The shift from hormone-sensitive PCa to CRPC is caused by cell adaptation to a microenvironment with low androgen levels. NEPC predominantly occurs after AR targeted therapies and is clinically recognized as a hormonal escape mechanism [23]. Several preclinical studies have shown that xenografts of AR-dependent LNCaP cells in castrated mice significantly increased the number of cancer cells positive for NE markers [24]. In vitro studies demonstrated that AR silencing using siRNA in LNCaP cells is sufficient to induce NSE expression and the outgrowth of a neuron-like phenotype [25]. 

EMT is involved in the progression towards a castration-resistant state, and may promote NED [23,26]. Indeed, overexpression of the EMT transcription factor Snail in LNCaP cells induced the expression of NE markers [27]. Moreover, the knockdown of Repressor Element-1 Silencing Transcription factor (REST), a transcriptional repressor for neuron-specific genes and a down-regulator of EMT [28], was shown to induce NED and enhance the expression of the EMT transcription factor Twist1 in LNCaP cells [28]. In human tissue samples, down-regulation of REST was observed in 50% of NEPC [29]. All these data provide evidence of a link between NED and EMT. However, the functional and molecular relationship between these processes in PCa cells has not been extensively explored. Interestingly, we demonstrated herein that Zeb1 is upregulated in CRPC-NE and is involved in the maintenance of NE markers in Enza-treated LNCaP cells. Indeed, Zeb1 inhibition is able to abolish Enza-induced NED. As previously demonstrated, Zeb1 is the pivotal EMT driver in human PCa cells, its expression increases with the stages of disease progression and is associated with decreased survival [8]. Despite Zeb1 induction, Enza does not impact E-cadherin and *n*-cadherin expression (data not shown) and unlike Zeb1, we observed that other EMT transcription factors, including Snail, Slug, and Twist, were down-regulated in Enza-treated LNCaP cells. A switch in the expression of EMT inducers was already described during the development of malignant melanoma. The authors showed that a reorganization of the EMT transcription factors network in favor of Zeb1 led to increased invasion properties and poorer prognosis [30]. These data suggest that Zeb1 may be acting outside of its role in EMT. 

A previous study showed that the expression of the Orai1 calcium channel is decreased in CRPC compared to CLC [31]. We reported herein a drastic reduction of Orai1 expression in CRPC-NE. Orai1 channel is involved in SOCE and CCE in several types of cancer cells [32,33]. The decreased expression of Orai1 in Enza-treated LNCaP could explain the decreasing of SOCE and CCE entries despite the increased expression of SK3. These results confirm the strong interaction between SK3 and the Orai1 channel in the management of calcium flux. Indeed, the reduction of expression of one ion channel that composes the onco-complex is sufficient to break the positive feedback loop between Orai1 and the SK3 channels [21,22]. In addition, the increase in SOCE observed 24 h following the Enza treatment could be explained by the increased expression of SK3, Orai2, and Orai3. Moreover, the induction of synaptophysin, NSE, and Zeb1 by Enza was decreased by the use of a CRACs channel inhibitor (GSK-7975A) (data not shown). A recent study shows that Orai2 and Orai3 channels are multimerised with Orai1 to allow calcium influx in response to low agonist concentration [34]. Our results are in agreement with other studies demonstrating a SOCE down-regulation in NE-differentiated LNCaP cells [35]. This deregulation may contribute to the anti-apoptotic potential of NE cells. Indeed, the down-regulation of Orai1, and consequently of SOCE, protects PCa cells from diverse apoptosis-inducing pathways [35].

The major result of this study is the up-regulation of SK3 in CRPC, including CRPC-NE. The SK3 channel was shown to form complexes with calcium channels, thereby leading to increased cytosolic calcium concentrations in cancer cells, and promoting the development of metastases [21]. We previously demonstrated that SK3 is a target gene of Zeb1 in PC3 cells [12] and this result was confirmed in LNCaP cells. Herein, we highlighted a positive feedback loop between Zeb1 and SK3 channel, involved in NED. Indeed, SK3 activation by CyPPA is able to strongly induce Zeb1 and NSE expression. By contrast, SK3 inhibition by Ohmline prevented the induction of Zeb1 and NE markers synaptophysin and NSE. The SK3/Zeb1 pathway is likely to be involved in the initial steps of NED. Indeed, SK3 inhibition is not able to reverse the process when the cells are well neurodifferentiated, after 1 week of the Enza treatment. This result suggests that, in order to abrogate NED, Ohmline should be combined with the anti-androgen at the very beginning of the treatment and in adjuvant therapies. The potential clinical interest of Ohmline was already demonstrated in mice with antimetastatic effects without any toxic effect [17,21]. Using ex vivo cultures of human PCa slices, which preserve both tumor microenvironment and cancer cell heterogeneity [18], we observed that Ohmline can prevent Enza-induced Zeb1. These preclinical data are an encouraging result, opening new therapeutic perspectives to avoid NED induced by AR-targeted treatments.

## 5. Conclusions

Treatment of patients with CRPC remains a significant clinical challenge. A better understanding of the mechanisms involved in NED, particularly after AR targeted therapies is needed to develop new therapeutic approaches. In this study, we identified the SK3 channel as a potential target of clinical interest in NEPC. A treatment combining antiandrogens with an inhibitor of SK3 such as Ohmline could constitute a strategy to avoid NED in CRPC.

## Figures and Tables

**Table 1:** Expression of Zeb1, SK3, Orai1 an Orai2 in prostate tissues (CLC: hormone naïve clinically-localized cancer; CRPC: Castration-resistant Prostate Cancer, adeno: adenocarcinoma; NE: neuroendocrine; NA: Not available.).

Variation	CLC (*n* = 220)	CRPC-Adeno (*n* = 50)	*p* (vs. CLC)	CRPC-NE (*n* = 16)	*p* (vs. CLC)
**Zeb1** − *% + cells (median, range)*	*NA: n = 6*	*NA: n = 2*	0.01	*NA*: *n = 2*	<0.0001
	− (*n* = 142)	− (*n* = 21)		− (*n* = 1)	
	+ (*n* = 72)	+ (*n* = 27)		+ (*n* = 13)	
	*10 (5−80)*	*10 (5−80)*		*40 (10−100)*	
**SK3**	*NA*: *n = 5*	*NA*: *n = 6*	<0.0001	*NA*: *n = 3*	<0.0001
	− (*n* = 158)	− (*n* = 7)		− (*n* = 5)	
	+ (*n* = 50)	+ (*n* = 15)		+ (*n* = 4)	
	++ (*n* = 7)	++ (*n* = 22)		++ (*n* = 4)	
**Orai1**	*NA: n = 26*	*NA: n = 3*	<0.0001	*NA: n = 2*	0.004
	− (*n* = 69)	− (*n* = 45)		− (*n* = 11)	
	+ (*n* = 79)	+ (*n* = 2)		+ (*n* = 3)	
	++ (*n* = 46)	++ (*n* = 0)		++ (*n* = 0)	
**Orai2**	*NA: n = 29*	*NA: n = 4*	0.02	*NA: n = 2*	0.0004
	− (*n* = 77)	− (*n* = 9)		− (*n* = 2)	
	+ (*n* = 92)	+ (*n* = 28)		+ (*n* = 5)	
	++ (*n* = 22)	++ (*n* = 9)		++ (*n* = 7)	

## Data Availability

The data presented in this study are available in this article (and supplementary material).

## Supplementary Materials

The following are available online at https://www.mdpi.com/article/10.3390/cancers13122947/s1, Figure S1: Enza decrease nuclear AR expression in LNCaP; Figure S2: Enza induces Zeb1, SK3 and NSE expression in C4-2 and C4-2B cell lines; Figure S3: Ohmline is unable to reverse NED in neurodifferenciated LNCaP cells; Table S1: Patients and tissues characteristics; Table S2: Primers used for quantitative real-time PCR; Table S3: siRNA sequences used for transfection assays; Table S4: Ca²^+^-free PSS composition used for Ca²^+^ measurements.

## Abbreviations

ADT	Androgen deprivation therapy
AM	Acetoxy methyl ester
AR	Androgen receptor
CCE	Constitutive calcium entry
CLC	Clinically localized cancer
CRPC	Castrated resistant prostate cancer
DHT	Dihydrotestosterone
DMSO	Dimethylsulfoxide
EMT	Epithelial-to-mesenchymal transition
Enza	Enzalutamide
HPRT	Hypoxanthine phosphoribosyltransferase)
KCNN3	Potassium Calcium-Activated Channel Subfamily *n* Member 3
LNCaP	Lymph Node Carcinoma of the Prostate
MTT	(3-(4,5-dimethylthiazol-2-yl)-2,5-diphenyl tetrazolium bromide
NE	Neuroendocrine
NED	Neuroendocrine differentiation
NEPC	Neuroendocrine prostate cancer
NSE	Neuron specific enolase
PCa	Prostate cancer
PSA	Prostate specific antigen
PSS	Physiological saline solution
REST	Repressor Element-1 Silencing Transcription factor
SOCE	Store-operated calcium entry
TBP	TATA-binding protein
Tg	Thapsigargine
TMA	Tissue Micro-array
TNM	Tumor Node Metastasis
Zeb1	Zinc finger enhancer binding protein 1
