# Supplementary material for: Zeb1 and SK3 Channel Are Up-Regulated in Castration-Resistant Prostate Cancer and Promote Neuroendocrine Differentiation
Source: Cancers (Basel). 2021 Jun 12;13(12):2947. doi: 10.3390/cancers13122947 (PMC8231145; doi:10.3390/cancers13122947)
Supplement: Supplementary file 1 [file cancers-13-02947-s001.zip › cancers-1227942-supplementary.pdf]

cancers

MDPI

Supplementary Materials: Zeb1 and SK3 Channel Are Up-Regulated in Castration-Resistant Prostate Cancer and Promote Neuroendocrine Differentiation

Fanny Bery, Mathilde Cancel, Maxime Guéguinou, Marie Potier-Cartereau, Christophe Vandier, Aurélie Chantôme, Roseline Guibon, Franck Bruyère, Gaëlle Fromont and Karine Mahéo

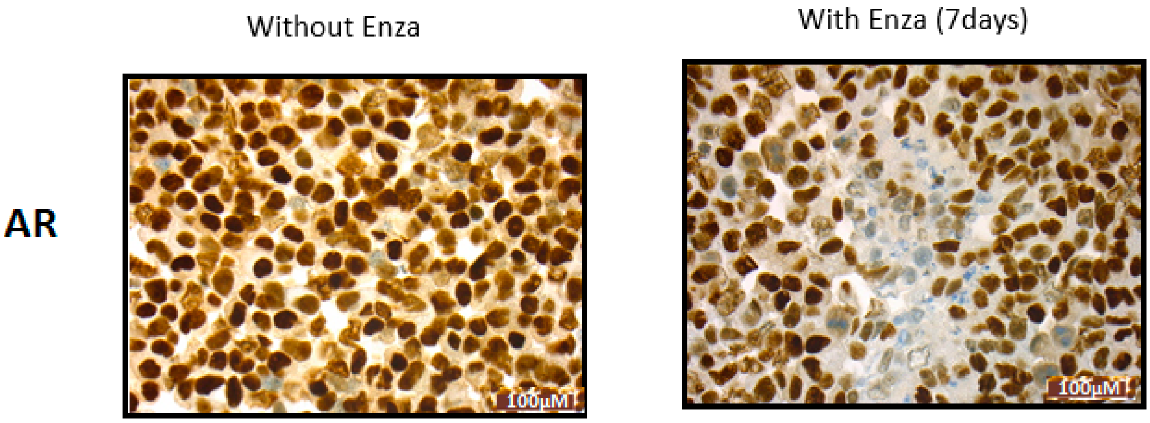

Figure S1. Enza decrease nuclear AR expression in LNCaP. LNCaP cells were treated or not for 7 days with Enza (10µM). AR protein staining was obtained by immunohistochemistry.

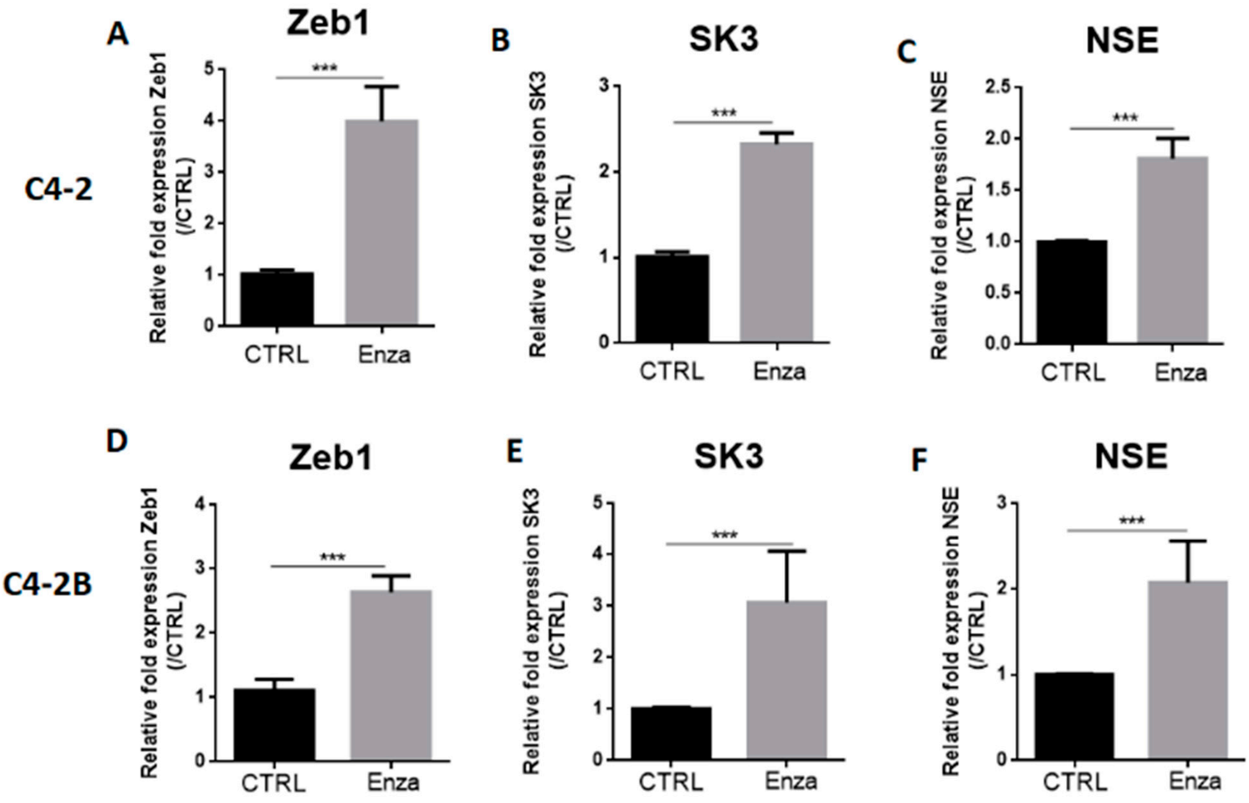

Figure S2. Enza induces Zeb1, SK3 and NSE expression in C4-2 and C4-2B cell lines. C4-2 and C4-2B cell lines were treated with Enza (10µM) for 1 week. qPCR results are normalized to control condition and are expressed as mean +/- S.E.M. The statistical differences are indicated: ***$p < 0{,}001$ (Mann–Whitney) $n = 3$.

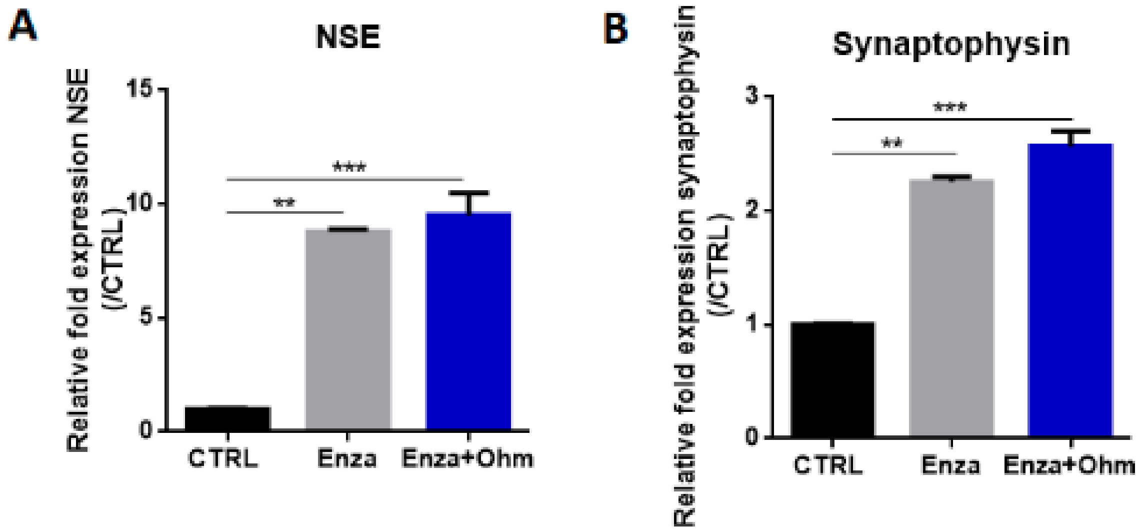

Figure S3. Ohmline is unable to reverse NED in neurodifferenciated LNCaP cells. LNCaP cells were treated with Enza (10µM) for 1 week and then co-treated with Enza (10µM) and Ohmline (1µM) for 96H. qPCR results are normalized to control condition and are expressed as mean ± S.E.M. The statistical differences are indicated: $^{**}p < 0{,}01$; $^{***}p < 0{,}001$ (Kruskal-Wallis, Post-test: Dunn) $n = 3$.

Table S1. Patients and tissues characteristics.

Groups	CLC (*n* = 220)	CRPC-adeno (*n* = 50)	CRPC-NE (*n* = 16)
**Age y, median (range)**	63 (46-75)	69 (48-91)	73 (52-87)
**PSA (ng/ml)**	7.4 (4.5-23)	26.9 (3.5-700)	4.1 (0.03-270)
**pTNM**			
pT2	108	NA	NA
pT3	112		
**ISUP score**			
**1**	49		
**2**	59	NA	NA
**3**	102		
**4/5**	10		

CLC: hormone-naïve clinically-localized cancer; CRPC: castration resistant prostate cancer; adeno: adenocarcinoma; NE: neuroendocrine; y: years; NA: not applicable.

Table S2. Primers used for quantitative real-time PCR

Name	Sequences 5'-3'
HPRT forward	5-TGACACTGGCAAAACAATGCA-3
HPRT reverse	5-GGTCCTTTTCACCAGCAAGCT-3
TBP forward	5-TGTATCCACAGTGAATCTTGGTTG-3
TBP reverse	5-GGTTCGTGGCTCTCTTATCCTC-3
Zeb1 forward	5-TGCACTGAGTGTGGAAAAGC-3
Zeb1 reverse	5-TGGTGATGCTGAAAGAGACG-3
SK3 exon5 forward	5-TGGACACTCAGCTCACCAAG-3
SK3 exon7 reverse	5-GTTCCATCTTGACGCTCCTC-3
NSE forward	5-TGGCTTTGCCCCCAATATCC-3
NSE reverse	5-TCTTTTCCGTGTAGCCAGCC-3
Synaptophysin forward	5-TGCCAACAAGACCGAGAGTG-3
Synaptophysin reverse	5-TTCGGCTGACGAGGAGTAGT-3
AR forward	5-AAGACGCTTCTACCAGCTCAC-3
AR reverse	5-TCCCAGAAAGGATCTTGGGCA-3
PSA forward	5-GGCAGCATTGAACCAGAGGA-3
PSA reverse	5-ATCACCCGAGCAGGTGCTTT-3
Snail forward	5-AATCCAGAGTTTACCTTCCAGCA-3
Snail reverse	5-TCCCAGATGAGCATTGGCAG-3
Slug forward	5-GAACTGGACACACATACAGTGAT-3
Slug reverse	5-ACAGTGATGGGGCTGTATGC-3
Twist forward	5-GCCAGGTACATCGACTTCCTCT-3
Twist reverse	5-TCCATCCTCCAGACCGAGAAGG-3
Vimentin forward	5-CCTTGAACGCAAAGTGGAATC-3
Vimention reverse	5-GACATGCTGTTCCTGAATCTGAG-3
MMP9 forward	5-ACGCACGACGTCTTCCAGTA-3
MMP9 reverse	5-CCACCTGGTTCAACTCACTCC-3
Orai1 forward	5-AGGTGATGAGCCTCAACGAG-3
Orai1reverse	5-CTGATCATGAGCGCAAACAG-3
Orai2 forward	5-GGCATTCGTATAAATGACCTGGTGG-3
Orai2 reverse	5-GTTGTGGATGTTGCTCACGG-3
Orai3 forward	5-CCACGTACCGGGAGTTCG-3
Orai3 reverse	5-ACTCGTGGTCACTCTCCAGC-3

Table S3. siRNA sequences used for transfection assays

Name	Sequences 5'-3'
siCTRL	5- GCCGACCAAUUCACGGCCG-3
siZeb1	5- GGCGGUAGAUGGUAAUGUA-3

Table S4. Ca^{2+}-free PSS composition used for cytosolic Ca^{2+} measurements (pH adjusted to 7.4 with NaOH).

Solution	Concentration (mM)
NaCl	140
MgCl2	2
KCl	4
D-Glucose	11.1
HEPES	10
EGTA	1
